# An Adapted Physical Activity Program for Adolescents with an Intellectual Disability: An International Classification of Functioning, Disability, and Health Perspective

**DOI:** 10.3390/life14101314

**Published:** 2024-10-16

**Authors:** Xin Shen, Peiying Huang, Bing Nie, Maolin Su, Dan Liu, Yin Guo, Lan Zheng

**Affiliations:** 1 Key Laboratory of Physical Fitness and Exercise Rehabilitation of Hunan Province, College of Physical Education, Hunan Normal University, Changsha 410012, China; 202270153062@hunnu.edu.cn (X.S.); 202220152990@hunnu.edu.cn (P.H.); 2 College of Physical Education, Hunan Normal University, No36 Lushan Road, Changsha 410012, China; 3 Changsha Special Education School, No190 Yanhang Road, Changsha 410012, China; 15973180020@163.com (B.N.); 17576007148@163.com (M.S.); 13874850099@163.com (D.L.)

**Keywords:** intellectual disabilities, fundamental motor skills, quality of life, physical fitness, International Classification of Functioning, Disability, and Health, adapted physical activity

## Abstract

The International Classification of Functioning, Disability, and Health (ICF) offers a comprehensive bio-psycho-social model for evaluating the multifaceted needs of individuals with disabilities. While its adoption in healthcare settings is widespread, its utilization within the domain of physical activity interventions, particularly for adolescents with intellectual disabilities (IDs), remains insufficiently explored. This study rigorously examines the efficacy of a 6-month ICF-based Adaptive Physical Activity (APA) intervention on the development of fundamental motor skills (FMSs), improvements in physical fitness (PF), and enhancements in quality of life (QoL) among adolescents with ID. A total of thirty-eight adolescents were randomly allocated into either an intervention group (IG), which participated in the tailored APA program, or a control group (CG), which received standard physical education. The findings demonstrated statistically significant improvements in the IG’s test of gross motor development (TGMD) total, locomotor, object control, and QOL scores relative to the CG (*p* < 0.001, η² = 0.330, 0.249, 0.224, and 0.439, respectively). Furthermore, substantial gains were observed in PF measures, including upper and lower limb strength as well as cardiorespiratory fitness (*p* < 0.001, η² = 0.254, 0.351, 0.176). Strong positive correlations were noted between FMS, PF, and QoL (r = 0.34–0.71, *p* < 0.05). This study underscores the importance of tailoring physical activity programs for adolescents with ID, offering insights into the relationships between FMS, PF, and QoL, and guidance for future interventions aimed at improving health outcomes in this population.

## 1. Introduction

The International Classification of Functioning, Disability, and Health (ICF) provides a framework that integrates the “bio-psycho-social” model to comprehensively describe the health and functionality of individuals with disabilities [1]. By defining physical functions and structures, activities, and participation, and explaining how these elements are influenced by health conditions and environmental factors, the ICF framework enhances precision in identifying rehabilitation needs and setting targeted intervention goals [2]. In recent years, the application of ICF has expanded beyond traditional clinical practice to encompass special education and disability assessments, enabling health professionals to systematically evaluate patient conditions, establish targeted therapeutic goals, and design tailored rehabilitation plans [3,4,5,6,7]. Moreover, the ICF theoretical models pertaining to sports participation exhibit a notable degree of development [8]. Carlin’s conceptual framework elucidates the intricate interactions between sports involvement and individuals with disabilities across personal, social, and policy dimensions, underscoring the framework’s potential to inform effective policy strategies [9]. For individuals with intellectual disabilities (IDs), the ICF core sets facilitate targeted assessments of specific health conditions and social participation challenges, thereby providing a framework for developing personalized interventions that effectively address their unique needs and enhance their overall well-being [10].

Individuals with ID often exhibit significant limitations in cognitive and adaptive functioning, impacting their conceptual, social, and practical skills [11]. This population frequently encounters a range of challenges, including motor, sensory, and communication impairments, as well as congenital heart conditions and delays in physical and mental development [12]. Individuals with ID engage in physical activities (PAs) less frequently than their typically developing peers, which contributes to a higher risk of health complications [13,14]. A combination of factors contributes to these barriers to physical activity. Developmental delays and poor motor skills hinder participation, while low self-efficacy and limited understanding of the health benefits of exercise further reduce motivation [15,16,17]. In addition to personal challenges, individuals with ID often face social and environmental obstacles, such as inadequate parental support, social isolation, limited access to suitable facilities, and unfavorable weather conditions [18,19]. Overweight and obesity are particularly prevalent in this population. A meta-analysis indicated that among children with ID, the prevalence rates for overweight, overweight–obesity, and obesity statuses are 15%, 30%, and 13%, respectively [20]. This high prevalence of obesity not only exacerbates their immediate health concerns but also significantly increases the likelihood of developing chronic conditions such as type 2 diabetes and cardiovascular diseases in adulthood [21,22,23,24]. Given these health challenges, it becomes evident that enhancing PA is crucial to mitigating the long-term health risks faced by individuals with ID [25,26,27].

PA serves as an effective and cost-efficient strategy for improving health outcomes in individuals with ID. Regular engagement in PA has been demonstrated to enhance physical fitness, encompassing aspects such as muscle strength and endurance, while also positively influencing overall quality of life (QoL) [28]. Moreover, participation in PA contributes to improvements in self-efficacy and mental health, thereby offering comprehensive benefits for this population [29]. Existing research has examined various forms of exercise, such as aerobic and resistance training, but often neglects the personal and environmental barriers faced by adolescents with ID in engaging in PA [30,31,32,33]. This oversight limits the effectiveness of these interventions. Late adolescence marks a crucial period for physical and motor development, characterized by increased adaptability and independence [34,35,36]. Addressing this developmental stage is critical for interventions aimed at supporting the transition to adulthood. This study employs the ICF framework to develop a more inclusive approach, aligning interventions with the practical needs of adolescents with ID and fostering sustainable health outcomes.

Fundamental motor skills (FMSs), which are foundations of more complex motor abilities, are critical for improving PA levels in children with ID [37,38]. Evidence increasingly supports the importance of FMS in the physical fitness (PF) of individuals. For example, research by Barnett and colleagues shows that children with less developed FMS tend to have lower PF [39]. Furthermore, FMS proficiency may correlate with enhanced overall QoL, as evidenced by superior physical, emotional, social, and academic outcomes in children who demonstrate higher levels of FMS compared to those with lower proficiency [40,41]. However, while the benefits of developing FMS of typically developing children are well documented, it remains unclear whether improvements in FMS performance translate into enhanced QoL and PF in adolescents with ID. Therefore, understanding the link between FMS and health outcomes, specifically in adolescents with ID, is crucial to advancing interventions that can effectively address both their motor skills and broader QoL.

This study aims to evaluate the effectiveness of an Adaptive Physical Activity (APA) program developed based on the ICF framework for adolescents with ID. The focus will not only be on improving motor skills but also on addressing broader social and environmental barriers to participation, as guided by the ICF framework. Based on the literature review, we propose the following hypotheses: (1) the ICF-based APA program will more effectively meet the needs of adolescents with ID compared to other exercise interventions; (2) this program will significantly improve their FMS performance and lead to enhancements in PF and QoL; (3) FMS of adolescents with ID will be strongly and positively correlated with PF and QoL.

## 2. Methods

### 2.1. Study Design

This 6-month randomized controlled trial (RCT) ran from February to August 2024. Each participant completed a baseline assessment before the intervention and a follow-up assessment after the intervention. Participants were randomly assigned to either the intervention group (IG) or control group (CG) using a computer-generated randomization sequence with a 1:1 allocation ratio. Using G*Power (version 3.1.9.7) for calculations, and assuming a significance level of 0.05 with an 80% power for a two-tailed test, the minimum number of participants necessary to achieve statistical significance in this pre–post, two-arm design is 17 per group. Accounting for a potential dropout rate of 10%, a total of 38 participants were needed for the study.

### 2.2. Participants

Forty participants were recruited from a specialized school in Changsha. Recruitment and eligibility screening were managed by the school’s physical education teachers. Inclusion criteria were as follows: (1) a diagnosis of mild to moderate ID (IQ range: 35–69); (2) an age range of 15–17 years; (3) the ability to independently engage in the physical activity intervention; and (4) informed consent and active cooperation from participants and their legal guardians. Exclusion criteria were as follows: (1) the presence of severe physical disabilities or medical conditions that contraindicated physical activity, such as severe heart conditions; (2) involvement in other exercise programs within the past six months, to control for prior exercise exposure that could impact the study; and (3) missing more than three intervention sessions, to ensure consistency and reliability of the intervention outcomes.

Participants provided informed consent with the approval of their parents or guardians. This randomized controlled trial (clinical registration: NCT06444659) was approved by the Ethics Committee of Hunan Normal University (Approval No. 301 08/05/2023) and conducted in accordance with the Declaration of Helsinki.

Before participating in this study, many adolescents reported varying degrees of involvement in sports and recreational activities. Most participants engaged primarily in physical education classes at school, which occur three times a week. Approximately 20% of the adolescents had prior experience with extracurricular sports activities, including basketball, soccer, and jump rope. Additionally, we conducted a brief educational background survey with the parents of the participants (see Table 1), which provides further context for understanding the factors that may influence physical activity participation and engagement in the intervention.

### 2.3. Intervention

**Figure 1:**
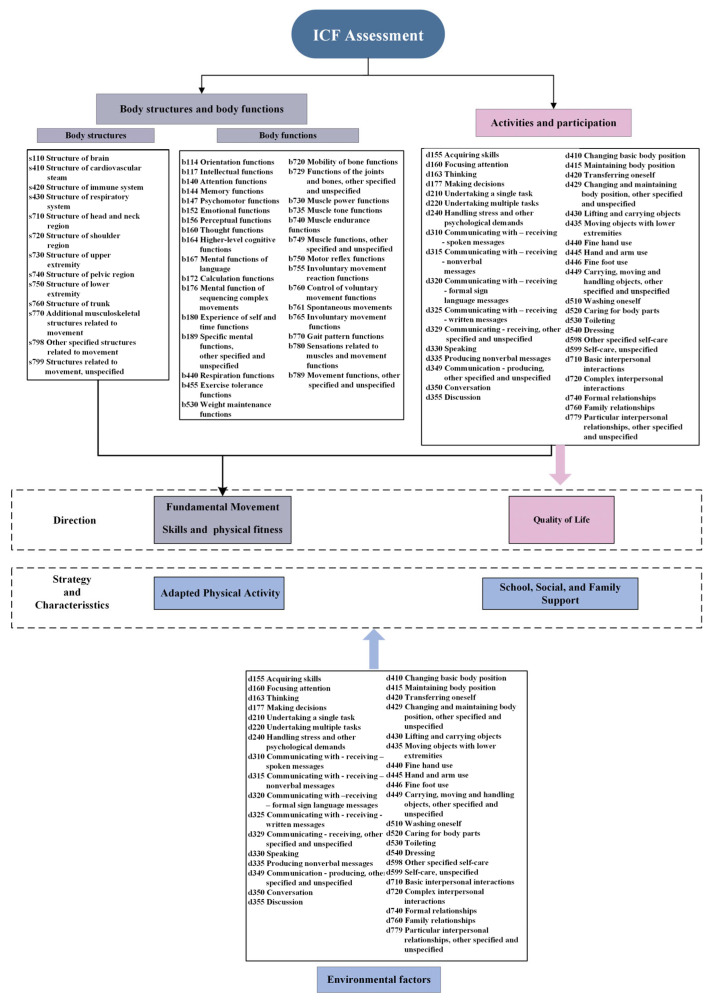
Rehabilitation goals and strategies developed based on the assessment of ICF categories.

Two professors and three experts in the field of special education conducted a comprehensive assessment of the participants based on the ICF framework. The assessment covered four areas: body structure, body function, activity and participation, and environmental factors. Evaluation criteria were adapted from the ICF core sets, specifically developed for children and adolescents with cerebral palsy, with each category scored on a scale from 0 to 4 and 8 to 9. A score of 0 indicates “no problem/difficulty”, while a score of 4 signifies a “complete problem/difficulty.” A score of 8 means “not specified”, and 9 indicates “not applicable” [42,43]. In the environmental factor category, a “+” symbol denotes a facilitating factor, while a “-” symbol indicates a barrier. After collecting all the necessary information, the research team assigned scores to each item, completing an ICF-based rehabilitation questionnaire to establish the goals and activities for the APA program. Specific categories and scoring criteria can be referenced in Figure 1.

The IG participated in a six-month APA program, meticulously designed by special education experts and implemented through three weekly sessions, each lasting 45 min. Prior to the intervention, a one-week adaptation phase was conducted to assist students with ID in acclimating to the training protocols and equipment. This phase emphasized a gradual adjustment to exercise intensity, ensuring that each activity was tailored to meet the individual physical capabilities and sensory needs of the students, thereby maximizing engagement and participation. The CG continued with their usual daily activities during the intervention period.

Based on ICF assessments, the limitations affecting the participation of adolescents with ID were identified, leading to several adaptations in the APA program. These adaptations included the following: (1) environmental modifications such as adjusting exercise space layouts, eliminating obstacles, soft-padding sharp corners, and providing adaptive equipment like adjustable-height jump boxes and modified resistance bands; and (2) sensory optimization measures that involved implementing soft lighting, adjusting sound levels, incorporating visual aids, providing tactile sensory items, and creating designated quiet areas. The program’s content selection was expanded to include a variety of activities aimed at fostering both participation and skill development. Key sports activities incorporated were basketball, soccer, badminton, and jump rope, complemented by fitness exercises such as quadrupedal crawling, obstacle courses, balance beam walking, and hurdle jumps. Exercise intensity was set at 40–80% of HRmax per ACSM guidelines for individuals with intellectual disabilities, with real-time feedback provided by Gymsmart (V 1.0; Gezhi Technology, Chengdu, China) equipment to allow for immediate adjustments, ensuring participants stayed within an effective range for meaningful participation and skill development.

Additionally, the program incorporated instructional strategies and parental involvement to enhance participation in PA among these adolescents. Teachers established clear and achievable goals, breaking down complex tasks into manageable steps to foster collaboration and interaction among students. For those with Down syndrome, strategies such as utilizing visual supports, providing consistent daily routines, and implementing positive reinforcement were employed to address emotional needs and offer timely feedback, thereby strengthening effective management. Furthermore, bi-weekly parent–child physical activity sessions fostered family involvement, equipping parents with the knowledge and skills to meet their children’s physical activity needs and enhancing the supportive role of families.

### 2.4. Measures

Adolescents with ID were assessed using the Test of Gross Motor Development-2 (TGMD-2). The TGMD-2 is a widely recognized tool for evaluating FMS of children, and its reliability and validity have been rigorously established in populations with ID [44]. This assessment included key motor skills such as running, jumping, throwing, and catching [45,46]. Although the TGMD-2 was initially developed for children aged 3 to 10, numerous studies have demonstrated its effectiveness and reliability in assessing adolescents with intellectual impairment [47,48,49]. To address the complex needs of adolescents with ID, assessments were conducted within familiar school environments. Prior to testing, a research assistant demonstrated each task twice for every participant. Participants then practiced the tasks twice with prompts before completing two assessment trials without prompts. The TGMD-2 assessments were administered by two special education experts with training in the relevant methodologies. To ensure accuracy, both assessors independently scored the TGMD-2 for typically developing adolescents before the testing, achieving a consistency rate of 90% over two consecutive days.

PF assessments were conducted in the following sequence: the body mass index (BMI), muscular strength and endurance [50,51] (evaluated via grip strength, a 30 s sit-to-stand test, and a 1 min sit-up test), flexibility [52] (assessed using the sit-and-reach test), and cardiorespiratory fitness [53] (measured by the 20 m PACER run). All tests were performed according to standardized protocols for both pre-intervention (baseline) and post-intervention evaluations. To accommodate the physical characteristics of adolescents with ID, adjustments were made to the assessment protocols. The sit-up test was modified to allow adolescents to avoid coming to a full sit-up position and experiment with various arm positions, while the grip strength test was conducted in a seated position [54]. In the 20 m PACER run, visual signal lights were employed at both the start and finish, while familiar physical education teachers provided pacing support and verbal cues throughout this specific test.

Considering the subjectivity of adolescents with ID, we selected the WHOQOL-DIS-ID scale to assess their QoL. This scale was developed to complement the WHOQOL-BREF and WHOQOL-100, specifically designed for individuals with ID [55]. Multiple studies have established its validity and reliability [56,57]. Participants responded to 12 items, each offering five possible answers.

### 2.5. Statistical Analyses

Descriptive statistics were reported as the mean ± standard deviation. All statistical analyses were carried out using SPSS version 23.0. To ensure the suitability of the data for analyses, the Shapiro–Wilk test was employed to assess normality, and the Levene test was used to verify the homogeneity of variances. A two-factor ANOVA was then applied to compare the dependent variables (FMS, PF, and QoL) between the IG and CG at baseline and after the intervention. Repeated measures analyses were performed to explore both inter-group and intra-group differences over time, with post hoc tests conducted for any significant findings. Additionally, Pearson correlation coefficients were calculated to examine the relationships between FMS, PF, and QoL. Correlation values of 0.1, 0.3, and 0.5 were interpreted as small, moderate, and large, respectively [58]. Gender, age, and BMI were controlled for in the regression models to ensure that they did not influence the outcomes related to QoL [59].

## 3. Results

### 3.1. Baseline Characteristics

**Figure 2:**
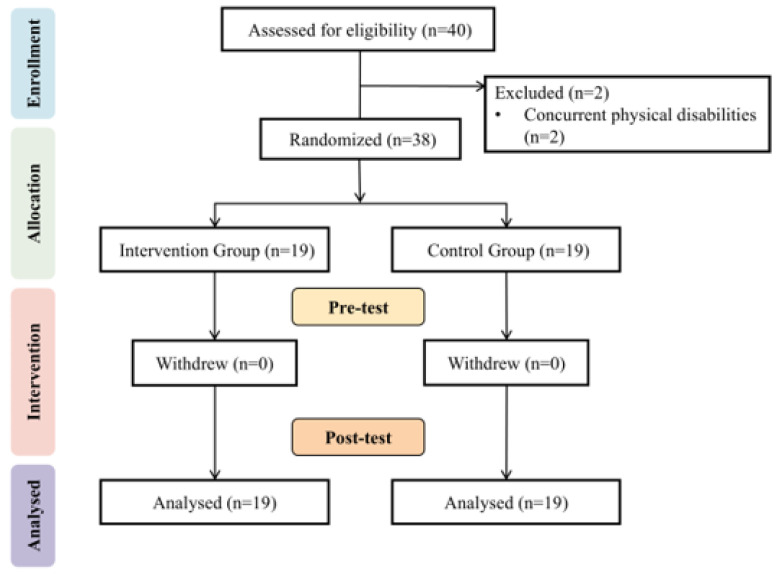
Flowchart of study participants’ experimental process.

As illustrated in the flowchart (Figure 2), two students were excluded from the preliminary screening due to concurrent physical disabilities. The final study cohort consisted of 24 students with a diagnosis of mild intellectual disability and 14 with moderate intellectual disability. Additionally, the cohort included three students with Down syndrome and three with autism spectrum disorder (Table 1).

### 3.2. Effect of APA on FMS and QoL

**Figure 3:**
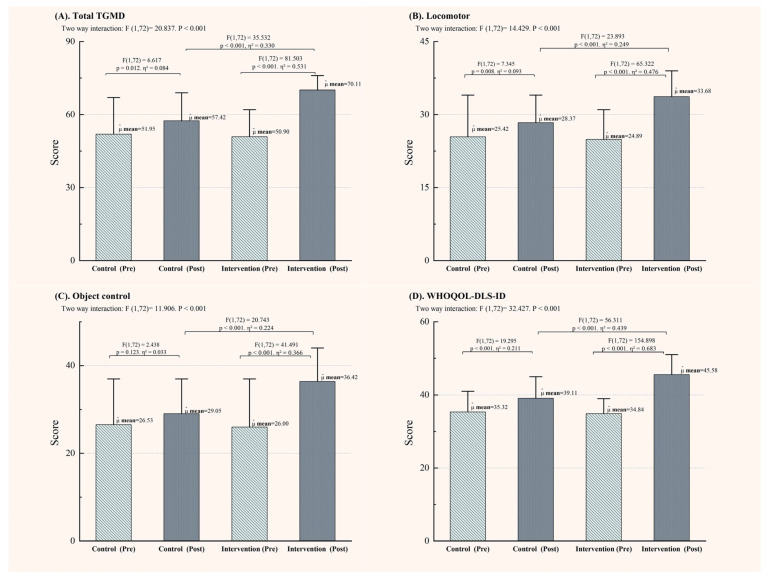
Scores of adolescents with intellectual disabilities on the TGMD-2 and WHOQoL-IDS-ID assessments. (**A**): Total scores of the TGMD-2; (**B**): Scores for locomotor skills; (**C**): Scores for control skills; (**D**): Scores for the WHOQOL-DLS-ID.

Figure 3 illustrates the TGMD-2 scoring results. After six months, although both the IG and CG showed improvements in the TGMD-2 total score, the IG demonstrated a significant increase of 12.69 points in the total score compared to the CG, with an improvement of 5.31 points in locomotor skills and 7.4 points in object control skills (all *p*-values less than 0.01).

Figure 3D presents the post-intervention QoL scores. After six months of physical activity intervention, all participants showed improvements in QoL scores (*p* < 0.001). However, the IG demonstrated more favorable outcomes compared to the CG, with an increase of 6.47 points.

### 3.3. Effect of APA on PF

**Figure 4:**
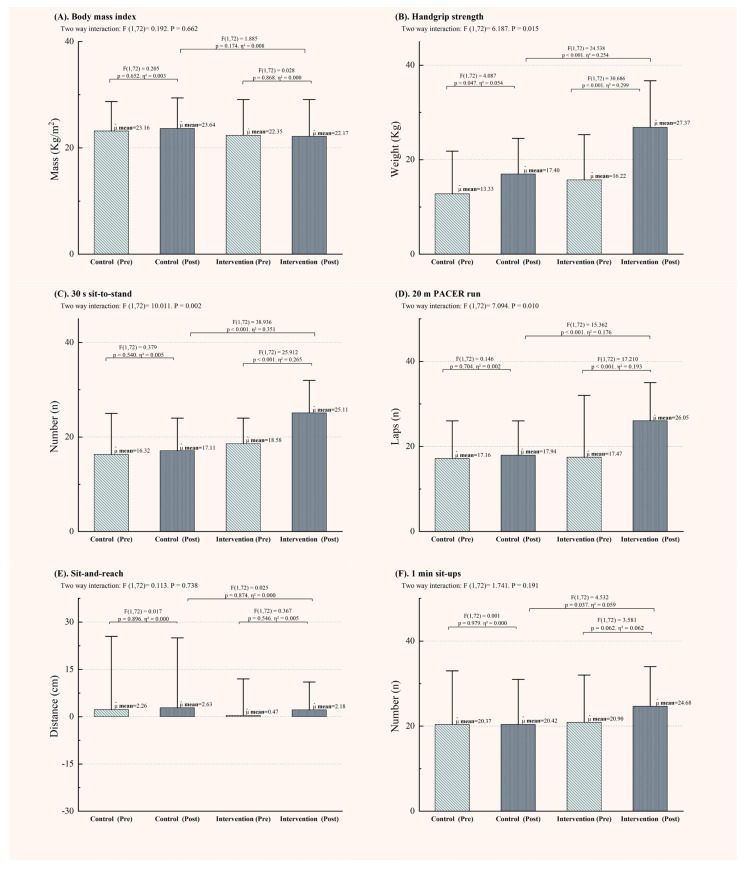
Performance Changes of Adolescents with Intellectual Disabilities in Physical Fitness Testing. (**A**): Changes for BMI; (**B**): Changes for Handgrip Strength; (**C**): Changes for the 30-Second Sit-to-Stand Test; (**D**): Changes for the 20-Meter PACER Run; (**E**): Changes for the Sit-and-Reach Test; (**F**): Changes for the 1-Minute Sit-Up Test.

Figure 4 illustrates the post-intervention PF outcomes, with significant interaction effects observed in the handgrip strength test (Figure 4B), the 30 s sit-to-stand test (Figure 4C), and the 20 m PACER run (Figure 4D). These findings further support the improvements in QoL. While both the CG and IG showed improvements in handgrip strength (measured in kilograms), the IG demonstrated a greater increase of 9.97 kg compared to the CG. Similarly, the IG improved by eight repetitions in the 30 s sit-to-stand test and by 8.11 laps in the 20 m PACER run, with all *p*-values less than 0.01. However, no significant changes were observed in BMI, the sit-and-reach test, or the 1 min sit-up test.

### 3.4. Correlation Analysis of FMS with QoL and PF

**Figure 5:**
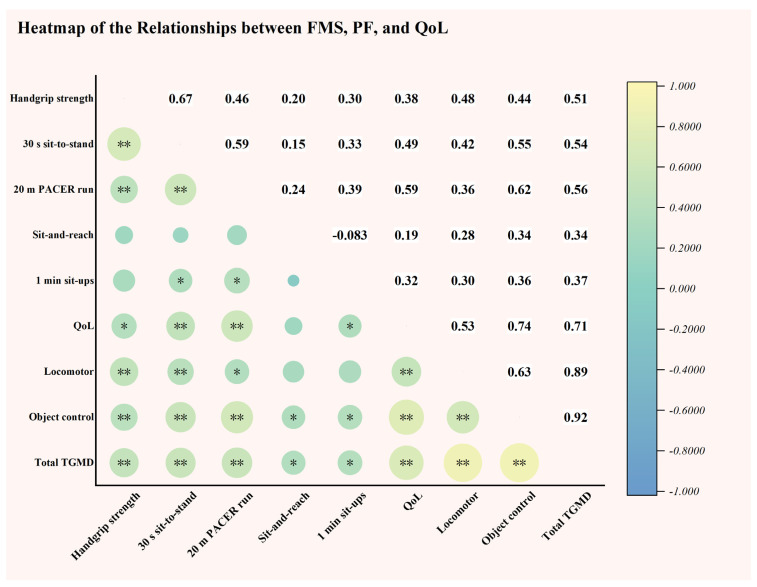
Heatmap of correlations among FMS, PF, and QoL. * *p* < 0.05; ** *p* < 0.001.

Figure 5 presents the correlation analysis among FMS, QoL, and PF in adolescents with ID. The results reveal a strong positive relationship between FMS and QoL (r = 0.71, *p* < 0.01), indicating that higher FMS scores were associated with improved QoL. FMS was also positively correlated with most PF components (r = 0.34–0.56, *p* < 0.05), suggesting that improvements in FMS were linked to enhancements in PF. QoL similarly shows positive correlations with PF variables (r = 0.32–0.59, *p* < 0.05), except for the sit-and-reach test, where no significant correlation was observed.

## 4. Discussion

The primary objective of this study was to investigate whether an APA program developed using the ICF framework could effectively address the unique needs of adolescents with ID. The findings revealed that a six-month APA intervention led to significant improvements in FMS, PF, and QoL among ID adolescents. These results support our research hypotheses, particularly Hypotheses 2 and 3, which posit that enhancing FMS positively impacts PF and QoL. Moreover, this study fills a critical gap in the existing literature by demonstrating that enhancing FMS of ID adolescents contributes to improvements in their PF and QoL. The successful implementation of the APA program underscores the utility of the ICF’s “bio-psycho-social” model in guiding the development of targeted rehabilitation goals and interventions for the ID population.

FMS is integral to various aspects of daily activities and is crucial for an individual’s physical, social, and psychological health [60]. This study found that after six months of implementing the APA program, participants demonstrated significant improvements in FMS, particularly in locomotor and object control skills. These results are consistent with existing research, which shows that targeted PA can substantially enhance FMS of children with ID [61,62,63]. Notably, while the existing literature primarily focuses on children, this study extends these findings to adolescents, demonstrating the broader applicability of FMS interventions across different age groups. Capio’s research suggests that incorporating strategies that create a familiar environment, which minimizes practice errors, into intervention programs is critical for effective FMS development in children with disabilities [64]. By reducing the number of errors during practice, these strategies decrease dependence on conscious processes and allow children to perform cognitively demanding secondary tasks without disrupting motor performance [65,66]. Our findings support this recommendation, as the APA program’s success in improving FMS and PF among adolescents with ID can be attributed to not only the structured PA but also the strategic environmental and social support modifications guided by the ICF framework. For instance, our program made significant efforts in redesigning exercise spaces, reducing sensory overload, and enhancing peer and parental support. These adjustments not only created a safe environment but also facilitated the development of long-term healthy behaviors among adolescents with ID.

The enhancement in FMS of adolescents with ID is expected to promote PF development, thus supporting Hypothesis 2. Specifically, the PF results indicated significant improvements in grip strength, the 30 s sit-to-stand test, and the 20 m PACER run post-intervention. These findings are consistent with other studies, suggesting that the program effectively improves muscular strength and cardiovascular endurance [26,67]. Despite notable improvements observed in various domains of PF, no significant changes were recorded in BMI, flexibility, or performance in the 1 min sit-up test. This absence of substantial progress in these specific areas may stem from the influence of uncontrolled external factors, such as dietary habits, during the intervention period. Research indicates that many children with ID exhibit poor eating behaviors due to a variety of factors [68], which could be a primary reason for the lack of significant alterations in BMI. Additionally, the specific nature of the PA may not have adequately targeted the components assessed by the flexibility and sit-up tests. For instance, the APA program primarily focused on dynamic and high-intensity activities, which might not have provided sufficient emphasis on exercises that enhance flexibility or core endurance. Future interventions could address these gaps by incorporating exercises specifically designed to target these areas.

Strong evidence links higher levels of FMS with better QoL and various functional outcomes, a relationship we believe extends to individuals with ID as well [40]. Cognitive–motor training, which integrates physical exercise with cognitive tasks, has been shown to significantly enhance cognitive function [69]. This improvement in cognitive function may contribute to the enhanced QoL observed in adolescents with ID; however, this aspect was not reflected in the results. Future studies should specifically explore the mechanisms underlying this relationship. Additionally, research suggests that individuals with higher FMS levels are more adept at participating in PA and social interactions, while those with lower FMS levels tend to experience increased fatigue and reduced PA participation, which can pose significant health risks [70,71]. Our study demonstrated that improvements in FMS were significantly associated with enhancements in PF, including increased grip strength, improved cardiorespiratory fitness, and better performance in the sit-to-stand test. These results suggest that as FMS improves, adolescents with ID are better equipped to engage in PA and manage daily tasks, thereby enhancing their overall PF. Furthermore, the advancement in FMS facilitates greater independence in navigating their environment, participating in recreational activities, and engaging in social interactions, all of which are crucial for improving QoL [72,73]. These results confirm our hypothesis that stronger FMS is strongly correlated with better PF and QoL. By addressing motor skill deficits, the APA program has enabled adolescents with ID to overcome previously challenging or unattainable daily activities. Such progress contributes to a more fulfilling and independent life, thereby significantly enhancing their QoL.

Tailored interventions for individuals with disabilities are essential for addressing their specific functional capacities [74,75,76], thereby optimizing the potential for meaningful and measurable improvements. Building on this principle, our study offers empirical evidence demonstrating that an exercise program designed using the ICF framework can lead to tangible health benefits for adolescents with ID. By employing the ICF assessment, a comprehensive understanding of these adolescents is achieved, allowing for the creation of customized educational training plans that enhance intervention effectiveness. Furthermore, we anticipate that the application of this methodology will extend beyond improving the QoL for individuals with ID, potentially benefiting other disability groups requiring personalized exercise interventions.

## 5. Study Limitations

While this study offers valuable insights, it is important to acknowledge several limitations. Firstly, the relatively small sample size may limit the applicability and generalizability of the findings. Secondly, the lack of assessment of family nutrition and physical activity habits represents a significant limitation; these factors should be considered and monitored in future research. Furthermore, the study did not explore the specific mechanisms linking improvements in FMS with changes in PF and QoL. Subsequent research should investigate these relationships to identify potential influencing factors. Another limitation of the current intervention is its primary focus on dynamic and high-intensity activities, which may not adequately address flexibility and core strength. The absence of significant improvements in these areas highlights the need for a more comprehensive exercise regimen that incorporates activities specifically designed to enhance flexibility and core endurance. Future programs should include a broader range of exercises to more effectively target these aspects.

## 6. Conclusions

This study investigated adolescents with ID aged 15–17 and found that the APA program, developed using the ICF framework, has a significant positive impact on improving FMS, PF, and QoL in these individuals. The findings partially support the hypothesis that enhancements in FMS are causally related to improvements in QoL and PF, addressing a notable gap in research on adolescents with ID. Furthermore, this research highlights the critical importance of incorporating both environmental and personal factors into physical activity programs for individuals with ID, as guided by the ICF framework.

The study’s results provide a strong foundation for future research aimed at expanding and refining these findings. Future studies should explore additional aspects of how physical activity affects individuals with ID, particularly in relation to long-term health outcomes and the sustainability of the observed benefits. By advancing our understanding of these relationships, we can improve the design and implementation of more effective, inclusive physical activity programs for individuals with ID.

## Figures and Tables

**Table 1:** Descriptive characteristics of participants.

Variables	Control Group (*n* = 19)	Intervention Group (*n* = 19)	Total (*n* = 38)
Age (years)	16.64 ± 0.84	16.43 ± 0.94	16.53 ± 0.88
Height (cm)	163.99 ± 8.11	166.30 ± 8.24	165.15 ± 8.62
Weight (kg)	62.33 ± 9.56	61.51 ± 10.11	61.92 ± 9.72
IQ	51.50 ± 5.75	53.43 ± 7.58	52.46 ± 6.67
BMI (kg/m^2^)	23.16 ± 2.79	22.35 ± 3.72	22.76 ± 3.27
	*n* (%)	*n* (%)	*n* (%)
Gender			
Boys	10 (52.6%)	9 (47.4%)	19 (50.0%)
Girls	9 (47.4%)	10 (52.6%)	19 (50.0%)
ID			
Mild (IQ: 55–69)	12 (63.2%)	12 (63.2%)	24 (63.2%)
Moderate (IQ: 35–54)	7 (37.8%)	7 (37.8%)	14 (36.8%)
Comorbidities			
Down syndrome	1 (5.3%)	1 (5.3%)	2 (5.2%)
Autism	2 (10.5%)	3 (15.8%)	5 (13.2%)
Intellectual disability only	16 (84.2%)	15 (78.9%)	31 (81.6%)
Mother’s Education Background			
No formal education	0 (0%)	0 (0%)	0 (0%)
Primary education	2 (10.5%)	1 (5.3%)	3 (7.9%)
Secondary education	10 (52.6%)	12 (63.2%)	22 (57.9%)
Higher education	7 (36.8%)	6 (31.5%)	13 (34.2%)
Mother’s Occupation			
Unemployed	0 (0%)	0 (0%)	0 (0%)
Manual labor	9 (47.3%)	7 (36.8%)	16 (42.1%)
Service sector	5 (26.3%)	7 (36.8%)	12 (31.6%)
Other	5 (26.3%)	5 (26.3%)	10 (26.3%)

ID: intellectual disability. BMI: body mass index. IQ: intelligence quotient.

## Data Availability

The datasets analyzed during the current study are available from the corresponding author on reasonable request.

## References

[1] World Health Organization (2007). International Classification of Functioning, Disability, and Health: Children & Youth Version: ICF-CY.

[2] McDougall J., Wright V., Rosenbaum P. (2010). The ICF model of functioning and disability: Incorporating quality of life and human development. Dev. Neurorehabil..

[3] Ehrmann C., Prodinger B., Stucki G., Cai W., Zhang X., Liu S., Liu S., Li J., Reinhardt J.D. (2018). ICF generic set as new standard for the system wide assessment of functioning in China: A multicentre prospective study on metric properties and responsiveness applying item response theory. BMJ Open.

[4] Stallinga H.A., Roodbol P.F., Annema C., Jansen G.J., Wynia K. (2014). Functioning assessment vs. conventional medical assessment: A comparative study on health professionals’ clinical decision-making and the fit with patient’s own perspective of health. J. Clin. Nurs..

[5] Rentsch H.P., Bucher P., Dommen N.I., Wolf C., Hefti H., Fluri E., Wenger U., Wälti C., Boyer I. (2003). The implementation of the International Classification of Functioning, Disability and Health (ICF) in daily practice of neurorehabilitation: An interdisciplinary project at the Kantonsspital of Lucerne, Switzerland. Disabil. Rehabil..

[6] Escorpizo R., Kostanjsek N., Kennedy C., Robinson Nicol M.M., Stucki G., Üstün T.B. (2013). Harmonizing WHO’s International Classification of Diseases (ICD) and International Classification of Functioning, Disability and Health (ICF): Importance and methods to link disease and functioning. BMC Public Health.

[7] Bagraith K.S., Strong J. (2013). The International Classification of Functioning, Disability and Health (ICF) can be used to describe multidisciplinary clinical assessments of people with chronic musculoskeletal conditions. Clin. Rheumatol..

[8] Clutterbuck G.L., Sousa Junior R.R., Leite H.R., Johnston L.M. (2024). The SPORTS Participation Framework: Illuminating the pathway for people with disability to enter into, participate in, and excel at sport. Braz. J. Phys. Ther..

[9] Carlin L., McPherson G., Davison R. (2024). The International Classification of Functioning Disability and Health Framework (ICF): A new approach to enhance sport and physical activity participation among people with disabilities in Scotland. Front. Sports Act. Living.

[10] Theil M.-M. (2021). Why ICF? Advantages of ICF in the clinical practice with regard to the medical care of people with mental health problems and intellectual disabilities. Eur. Psychiatry.

[11] Schalock R.L., Borthwick-Duffy S.A., Bradley V.J., Buntinx W.H., Coulter D.L., Craig E.M., Gomez S.C., Lachapelle Y., Luckasson R., Reeve A. (2010). Intellectual Disability: Definition, Classification, and Systems of Supports.

[12] Nakken H., Vlaskamp C. (2007). A need for a taxonomy for profound intellectual and multiple disabilities. J. Policy Pract. Intellect. Disabil..

[13] Borland R., Hu N., Tonge B., Einfeld S., Gray K. (2020). Participation in sport and physical activity in adults with intellectual disabilities. J. Intellect. Disabil. Res..

[14] Lin P.-Y., Lin L.-P., Lin J.-D. (2010). Hypertension, hyperglycemia, and hyperlipemia among adolescents with intellectual disabilities. Res. Dev. Disabil..

[15] Ptomey L.T., Willis E.A., Greene J.L., Danon J.C., Chumley T.K., Washburn R.A., Donnelly J.E. (2017). The Feasibility of Group Video Conferencing for Promotion of Physical Activity in Adolescents With Intellectual and Developmental Disabilities. Am. J. Intellect. Dev. Disabil..

[16] Barr M., Shields N. (2011). Identifying the barriers and facilitators to participation in physical activity for children with Down syndrome. J. Intellect. Disabil. Res..

[17] Downs S.J., Knowles Z.R., Fairclough S.J., Heffernan N., Whitehead S., Halliwell S., Boddy L.M. (2014). Exploring teachers’ perceptions on physical activity engagement for children and young people with intellectual disabilities. Eur. J. Spec. Needs Educ..

[18] Njelesani J., Leckie K., Drummond J., Cameron D. (2015). Parental perceptions of barriers to physical activity in children with developmental disabilities living in Trinidad and Tobago. Disabil. Rehabil..

[19] Yu S., Wang T., Zhong T., Qian Y., Qi J. (2022). In Barriers and facilitators of physical activity participation among children and adolescents with intellectual disabilities: A scoping review. Healthcare.

[20] Maiano C., Hue O., Morin A.J.S., Moullec G. (2016). Prevalence of overweight and obesity among children and adolescents with intellectual disabilities: A systematic review and meta-analysis. Obes. Rev..

[21] Emerson E., Robertson J., Baines S., Hatton C. (2016). Obesity in British children with and without intellectual disability: Cohort study. BMC Public Health.

[22] Melville C.A., Hamilton S., Hankey C.R., Miller S., Boyle S. (2007). The prevalence and determinants of obesity in adults with intellectual disabilities. Obes. Rev..

[23] Oppewal A., Hilgenkamp T.I., van Wijck R., Schoufour J.D., Evenhuis H.M. (2014). Physical fitness is predictive for a decline in daily functioning in older adults with intellectual disabilities: Results of the HA-ID study. Res. Dev. Disabil..

[24] Choi E., Park H., Ha Y., Hwang W.J. (2012). Prevalence of overweight and obesity in children with intellectual disabilities in Korea. J. Appl. Res. Intellect. Disabil..

[25] Thevarajah A., Wallen M., Imms C., Lonsdale C., Carey J.J., Froude E.H. (2022). Impact of adapted bicycle riding on outcomes for children and adolescents with disabilities: A systematic review. Dev. Med. Child Neurol..

[26] Wang A., Bu D., Yu S., Sun Y., Wang J., Lee T.C.T., Baker J.S., Gao Y. (2022). Effects of a school-based physical activity intervention for obesity, health-related physical fitness, and blood pressure in children with intellectual disability: A randomized controlled trial. Int. J. Environ. Res. Public Health.

[27] LeMura L.M., Maziekas M.T. (2002). Factors that alter body fat, body mass, and fat-free mass in pediatric obesity. Med. Sci. Sports Exerc..

[28] Jacob U.S., Pillay J., Johnson E., Omoya O.T., Adedokun A.P. (2023). A systematic review of physical activity: Benefits and needs for maintenance of quality of life among adults with intellectual disability. Front. Sports Act. Living.

[29] Kapsal N.J., Dicke T., Morin A.J.S., Vasconcellos D., Maïano C., Lee J., Lonsdale C. (2019). Effects of Physical Activity on the Physical and Psychosocial Health of Youth With Intellectual Disabilities: A Systematic Review and Meta-Analysis. J. Phys. Act. Health.

[30] Ogg-Groenendaal M., Hermans H., Claessens B. (2014). A systematic review on the effect of exercise interventions on challenging behavior for people with intellectual disabilities. Res. Dev. Disabil..

[31] Obrusnikova I., Firkin C.J., Farquhar W.B. (2022). A systematic review and meta-analysis of the effects of aerobic exercise interventions on cardiorespiratory fitness in adults with intellectual disability. Disabil. Health J..

[32] Bodde A.E., Seo D.-C. (2009). A review of social and environmental barriers to physical activity for adults with intellectual disabilities. Disabil. Health J..

[33] Bossink L.W., van der Putten A.A., Vlaskamp C. (2017). Understanding low levels of physical activity in people with intellectual disabilities: A systematic review to identify barriers and facilitators. Res. Dev. Disabil..

[34] Zarrett N., Eccles J. (2006). The passage to adulthood: Challenges of late adolescence. New Dir. Youth Dev..

[35] Brod’áni J., Lipárová S., Král’ M. (2016). The interaction of physical activity and the lif e quality of students in mid and late adolescence. Phys. Act. Rev..

[36] Howie E., McVeigh J., Smith A., Zabatiero J., Bucks R., Mori T., Beilin L., Straker L. (2020). Physical activity trajectories from childhood to late adolescence and their implications for health in young adulthood. Prev. Med..

[37] Barnett L.M., Stodden D., Cohen K.E., Smith J.J., Lubans D.R., Lenoir M., Iivonen S., Miller A.D., Laukkanen A., Dudley D. (2016). Fundamental movement skills: An important focus. J. Teach. Phys. Educ..

[38] Lubans D.R., Morgan P.J., Cliff D.P., Barnett L.M., Okely A.D. (2010). Fundamental movement skills in children and adolescents: Review of associated health benefits. Sports Med..

[39] Barnett L.M., Van Beurden E., Morgan P.J., Brooks L.O., Beard J.R. (2008). Does childhood motor skill proficiency predict adolescent fitness?. Med. Sci. Sports Exerc..

[40] Xie S., Zhou Y., Yin Y., Shao R., Fang L., Shao W. (2023). Effects of fundamental movement skills on health-related quality of life in Chinese school-age children: The mediating role of physical fitness level. Front. Public Health.

[41] Redondo-Tebar A., Fatouros I.G., Martinez-Vizcaino V., Ruíz-Hermosa A., Notario-Pacheco B., Sanchez-Lopez M. (2021). Association between gross motor competence and health-related quality of life in (pre) schoolchildren: The mediating role of cardiorespiratory fitness. Phys. Educ. Sport Pedagog..

[42] Yang L., Li S.S., Zhang G.Y., Wang M.M., Chen G.X., Zhu D.N. (2021). Effect of rehabilitation treatment based on the ICF-CY Core Sets on activities of daily living in children with cerebral palsy: A prospective randomized controlled study. Zhongguo Dang Dai Er Ke Za Zhi.

[43] Schiariti V., Selb M., Cieza A., O’Donnell M. (2015). International Classification of Functioning, Disability and Health Core Sets for children and youth with cerebral palsy: A consensus meeting. Dev. Med. Child Neurol..

[44] Griffiths A., Toovey R., Morgan P.E., Spittle A.J. (2018). Psychometric properties of gross motor assessment tools for children: A systematic review. BMJ Open.

[45] Hong-xia M. (2007). Study of the credibility and validity of the test of gross motor development of children. J. Phys. Educ..

[46] Valentini N.C. (2012). Validity and reliability of the TGMD-2 for Brazilian children. J. Mot. Behav..

[47] Chun H., Yun J., Hautala R., Nam D. (2002). Cross-validation of Test of Gross Motor Development for youth with mental retardation. Res. Q. Exerc. Sport.

[48] Foley J.T., Harvey S., Chun H.-J., Kim S.-Y. (2008). The relationships among fundamental motor skills, health-related physical fitness, and body fatness in South Korean adolescents with mental retardation. Res. Q. Exerc. Sport.

[49] Wang T., Qian Y., Zhong T., Qi J. (2022). Associations between Fundamental Movement Skills and Moderate-to-Vigorous Intensity Physical Activity among Chinese Children and Adolescents with Intellectual Disability. Int. J. Environ. Res. Public Health.

[50] Lee S.Y. (2021). Handgrip Strength: An Irreplaceable Indicator of Muscle Function. Ann. Rehabil. Med..

[51] Skowronski W., Horvat M., Nocera J., Roswal G., Croce R. (2009). Eurofit special: European fitness battery score variation among individuals with intellectual disabilities. Adapt. Phys. Act. Q..

[52] Frey G.C., Chow B. (2006). Relationship between BMI, physical fitness, and motor skills in youth with mild intellectual disabilities. Int. J. Obes..

[53] Wouters M., Evenhuis H.M., Hilgenkamp T.I. (2017). Systematic review of field-based physical fitness tests for children and adolescents with intellectual disabilities. Res. Dev. Disabil..

[54] Winnick J.P., Short F.X. (1998). Project Target: Criterion-Referenced Physical Fitness Standards for Adolescents with Disabilities. Final Report.

[55] Power M.J., Green A.M. (2010). Development of the WHOQOL disabilities module. Qual. Life Res..

[56] Sorkhi N., Akbarzade I., Nedjat S., Khosravi M., Nazemipour M., Memari A.H., Mansournia M.A. (2024). Validity and reliability of the persian version of the world health organization quality of life disabilities module. J. Intellect. Disabil..

[57] Bredemeier J., Wagner G.P., Agranonik M., Perez T.S., Fleck M.P. (2014). The World Health Organization Quality of Life instrument for people with intellectual and physical disabilities (WHOQOL-Dis): Evidence of validity of the Brazilian version. BMC Public Health.

[58] Cohen J. (2013). Statistical Power Analysis for the Behavioral Sciences.

[59] Haraldstad K., Christophersen K.A., Eide H., Nativg G.K., Helseth S. (2011). Predictors of health-related quality of life in a sample of children and adolescents: A school survey. J. Clin. Nurs..

[60] Bremer E., Cairney J. (2018). Fundamental movement skills and health-related outcomes: A narrative review of longitudinal and intervention studies targeting typically developing children. Am. J. Lifestyle Med..

[61] Zhang L., Zhu X., Haegele J.A., Wang D., Wu X. (2021). Effects of a one-year physical activity intervention on fundamental movement skills of boys with severe intellectual disabilities. Res. Dev. Disabil..

[62] Regaïeg G., Kermarrec G., Sahli S. (2020). Designed game situations enhance fundamental movement skills in children with Down syndrome. J. Intellect. Disabil. Res..

[63] Hocking J., McNeil J., Campbell J. (2016). Physical therapy interventions for gross motor skills in people with an intellectual disability aged 6 years and over: A systematic review. JBI Evid. Implement..

[64] Capio C.M., Poolton J., Sit C., Eguia K., Masters R. (2013). Reduction of errors during practice facilitates fundamental movement skill learning in children with intellectual disabilities. J. Intellect. Disabil. Res..

[65] Maxwell J.P., Masters R.S.W., Kerr E., Weedon E. (2001). The implicit benefit of learning without errors. Q. J. Exp. Psychol..

[66] Poolton J.M., Masters R.S.W., Maxwell J.P. (2005). The relationship between initial errorless learning conditions and subsequent performance. Hum. Mov. Sci..

[67] Elmahgoub S.M., Lambers S., Stegen S., Van Laethem C., Cambier D., Calders P. (2009). The influence of combined exercise training on indices of obesity, physical fitness and lipid profile in overweight and obese adolescents with mental retardation. Eur. J. Pediatr..

[68] Wang J., Gao Y., Kwok H.H.M., Huang W.Y.J., Li S., Li L. (2018). Children with Intellectual Disability Are Vulnerable to Overweight and Obesity: A Cross-Sectional Study among Chinese Children. Child. Obes..

[69] Gronek P., Adamczyk J.A.N., Celka R., Gronek J. (2021). Cognicise—A new model of exercise. Trends Sport Sci..

[70] Piek J.P., Dawson L., Smith L.M., Gasson N. (2008). The role of early fine and gross motor development on later motor and cognitive ability. Hum. Mov. Sci..

[71] Hands B. (2008). Changes in motor skill and fitness measures among children with high and low motor competence: A five-year longitudinal study. J. Sci. Med. Sport.

[72] Schott N., Holfelder B. (2015). Relationship between motor skill competency and executive function in children with D own’s syndrome. J. Intellect. Disabil. Res..

[73] Jaakkola T., Hillman C., Kalaja S., Liukkonen J. (2015). The associations among fundamental movement skills, self-reported physical activity and academic performance during junior high school in Finland. J. Sports Sci..

[74] Vlaskamp C., van der Putten A. (2009). Focus on interaction: The use of an Individualized Support Program for persons with profound intellectual and multiple disabilities. Res. Dev. Disabil..

[75] Cavaggioni L., Trecroci A., Tosin M., Iaia F.M., Alberti G. (2019). Individualized dry-land intervention program for an elite Paralympic swimmer. J. Sports Med. Phys. Fit..

[76] Seong Y., Wehmeyer M.L., Palmer S.B., Little T.D. (2015). Effects of the Self-Directed Individualized Education Program on Self-Determination and Transition of Adolescents With Disabilities. Career Dev. Transit. Except. Individ..

